# Microbial metabolite 5-formamidoimidazole-4-carboxamide ribotide targets *METTL1* to inhibit m7G modification of *BRCA1* mRNA to inhibit high-grade serous ovarian cancer

**DOI:** 10.1186/s10020-025-01396-y

**Published:** 2025-12-22

**Authors:** Lixing Chen, Sili He

**Affiliations:** Department of Gynecology, The Third Xiangya Hospital of Central South University, Central South University, Changsha, Hunan China

**Keywords:** High-grade serous ovarian cancer, *METTL1*, m7G, *BRCA1*, Microbial metabolite

## Abstract

**Background:**

This study explored the impact of vaginal microbes, metabolites, and *METTL1*-mediated m7G modification of *BRCA1* mRNA on High-Grade Serous Ovarian Cancer (HGSOC).

**Methods:**

*METTL1* and *BRCA1* expression levels were assessed via bioinformatics, Western blotting, and RT-qPCR. Their interaction was studied using RNA co-immunoprecipitation and RNA pull-down assays. The functions and mechanisms of *METTL1* and *BRCA1* in HGSOC were investigated through CCK-8 assays, flow cytometry, transwell migration assays, and nude mouse xenograft models. We analyzed vaginal microbial and metabolite differences in HGSOC patients with varying *BRCA1* expression using 16 S rRNA sequencing and liquid chromatography. Associations were evaluated with Spearman correlation and heat maps, while molecular docking assessed key metabolite binding to *METTL1*. The roles and interactions of selected metabolites with *METTL1/BRCA1* in HGSOC were validated through in vivo and in vitro experiments.

**Results:**

In HGSOC, both *METTL1* and *BRCA1* were up-regulated. *METTL1* enhanced *BRCA1* expression via m7G modification, boosting cell proliferation and tumor growth. Elevated *BRCA1* levels were associated with changes in vaginal microbiota, particularly increased Lactobacillus, and alterations in metabolic pathways. Correlation analysis indicated that Lactobacillus was significantly negatively correlated with 5-formamidoimidazole-4-carboxamide ribotide, inosine, cobalt-precorrin-7, and uridine, but positively correlated with L-lysine. The strongest correlation was with 5-formamidoimidazole-4-carboxamide ribotide. Molecular docking showed that this compound binds strongly to *METTL1*. Functional tests demonstrated that it inhibits HGSOC cell proliferation and tumor growth by disrupting *METTL1*-mediated m7G modification of *BRCA1*. Overexpression of *METTL1* or *BRCA1* negated its anti-tumor effects.

**Conclusion:**

The vaginal microbial metabolite 5-formamidoimidazole-4-carboxamide ribotide reduces *BRCA1* expression and slows HGSOC progression by modifying *BRCA1* m7G through *METTL1*, suggesting its potential as an HGSOC treatment.

**Supplementary Information:**

The online version contains supplementary material available at 10.1186/s10020-025-01396-y.

## Introduction

High-grade serous ovarian carcinoma (HGSOC), the most prevalent type of ovarian cancer (OC), is responsible for 70%−80% of OC-related deaths (Senkowski et al. 2023). Molecularly, around 50% of HGSOC patients exhibit deficiencies in homologous DNA recombination and inactivation of DNA damage/repair pathways (Janíková et al. 2021). The introduction of polyADP-ribose polymerase inhibitors (PARPi) has shown improved outcomes in women with *BRCA1/2* deficient or homologous recombination (HR) deficient HGSOC (Bound et al. 2022; Li and Zou 2024). However, the development of acquired drug resistance poses a significant challenge in PARPi treatment (Gralewska et al. 2024). Recent research has highlighted the link between the collective genome of vaginal microbial species and gynecological diseases, such as bacterial vaginosis and cervical cancer (Paola et al. 2017; Peremykina et al. 2024). Still, their involvement in OC, particularly HGSOC, remains understudied. It is recognized that dysregulation of microbiota in various sites, including tumor tissues, intestines, vagina, and serum, is a hallmark of OC (Sipos et al. 2021). Therefore, investigating the interplay between the vaginal microenvironment and HGSOC is crucial for devising strategies to overcome PARPi resistance and develop novel and effective treatments for HGSOC.

Breast cancer susceptibility gene 1 (*BRCA1*) is a known predisposing gene for breast and ovarian cancers, and its methylation status is linked to homologous recombination defects (Catteau and Morris 2002; Kalachand et al. 2020). Approximately 10%−15% of HGSOC cases are reported to have *BRCA1* promoter methylation (Lønning et al. 2022). Recent findings on *BRCA1* hypermethylation suggest that this epigenetic change may have a significant role in tumorigenesis (Ruscito et al. 2014). Methyltransferase-like protein 1 (*METTL1*) is a crucial enzyme involved in N-7 methylguanosine (m7G) methylation modification (Ruiz-Arroyo et al. 2023), but its role in OC remains largely unexplored. In a study investigating the development of an OC prognosis model, *METTL1* showed significant correlations with overall survival, cancer status, and immune cell profiles (Zheng et al. 2022). These observations led us to hypothesize that *METTL1* may regulate *BRCA1* expression via m7G modification, thereby influencing processes involved in HGSOC.

Prior studies have shown that disruptions in the female reproductive tract microbiome could lead to metabolic imbalances in amino acids, carbohydrates, and lipids, which are considered risk factors for gynecological diseases (Cao et al. 2024). Variations in the vaginal microbiome and metabolites have been observed between individuals with cervical HPV infection and those without infection (Borgogna et al. 2020; Shannon et al. 2017). It is hypothesized that changes in the vaginal microenvironment, stemming from shifts in vaginal microflora and metabolic disorders, may contribute to the development of persistent HPV-mediated cervical cancer and genital inflammation (Ilhan et al. 2019). Additionally, Previous research indicated that the use of antibiotics could impact tumor progression in mouse models of HGSOC with inactivation of *BRCA1*, Trp53, Rb1, and Nf1, potentially due to changes in the composition of intestinal and vaginal microbiota (Chen et al. 2021). Clinical studies have also found a significant association between *BRCA1* mutations and type O cervicovaginal microbiota when compared to controls with wild-type *BRCA1* (Nené et al. 2019). A conditioned medium of Enterococcus faecalis (Pourmollaei et al. 2023; Sharma et al. 2021) and proteins secreted by three types of Enterococcus extracted (Sharma et al. 2018) from vaginal secretion cultures isolated from healthy women are known to selectively inhibit the proliferation of human cancer cell lines in a dose-dependent manner. However, it remains unclear whether there is a correlation between *BRCA1* expression and alterations in the vaginal microbiota or metabolites, necessitating further investigation to elucidate the potential relationship with HGSOC.

Given the pivotal role of *BRCA1* and the significance of variations in the vaginal microenvironment in cancer, we are keen on delving deeper into the impact of differential *BRCA1* expression on the vaginal microenvironment in HGSOC. Our hypothesis posits that *BRCA1*-associated vaginal microbiota or metabolites may interact with the *METTL1/BRCA1* axis and partake in the progression of HGSOC. To explore this hypothesis, we plan to employ experimental techniques such as microbiome analysis, metabolomics, molecular docking, cell biology assays, RIP, RNA pull-down experiments, and HGSOC mouse models to examine its mechanism of action comprehensively. This endeavor aims to establish a novel theoretical foundation for advancing the treatment of HGSC.

## Materials and methods

### Bioinformatics analysis

We use The TCGA (The Cancer Genome Atlas) database (https://portal.gdc.cancer.gov/) to download and collect ovarian Cancer-related data and The corresponding clinical information, and GTEx (Genotype - Tissue Expression) database (https://www.gtexportal.org/) to collect normal Tissue sample data. Differential expression genes in ovarian cancer were identified by combining the TCGA database with the GTEx database. In this study, 427 tumors and 88 normal tissues were obtained from the TCGA dataset and GTEx, respectively. Wilcoxon test was used to compare *BRCA1* and *METTL1* expression in Normal and Tumor. The cutoff of *BRCA1* and *METTL1* expression groups was logFC > log2(1.5) & *p* < 0.05. Using the R software package “survminer”, Kaplan-Meier survival plots were used to estimate the OS between *BRCA1* and *METTL1* expression groups, respectively. Cox regression for survival analysis was performed by the R software package “survival”. A time-dependent receiver operating characteristic (ROC) curve was plotted using the R software package “timeROC”. The cutoff genes of *BRCA1* and METTL1 up-regulation were logFC > log2(1.5) & *p* < 0.05. Based on the R software package “VennDigram”, the intersection genes of *METTL1* and *BRCA1* with high expression in OV were analyzed. Intersection genes were obtained and used for functional enrichment analysis. Gene Ontology Enrichment Analysis (GO) and Kyoto Encyclopedia of Genes and Genomes (KEGG) analyses were performed based on gene sets defined in the MSIADB (Molecular Signatures Database) database.

In addition, we used the GEPIA database (http://gepia.cancer-pku.cn/detail.php) to analyze the correlation between METTL1 and BRCA1. Based on the RBP-mRNA interactions interface in CLIP-seq Data (https://rnasysu.com/encori/rbpClipRNA.php?source=mRNA), we analyzed the interaction between = METTL1 and BRCA1 protein. The m7GHub database (http://www.rnamd.org/m7GHub2/index.html) was used to predict the likelihood of BRCA1 mRNA m7G modification.

### Collection of vaginal swab samples

Fourteen patients with metastatic or primary HGSOC participated in the study after giving informed consent. During surgery, cancerous and adjacent tissues were collected from 8 patients for Western blot analysis to measure *METTL1* and *BRCA1* expression levels. Additionally, to study the link between cervicovaginal microbiome-metabolites and *BRCA1* expression, HGSOC patients were divided into high (*N* = 6) and low (*N* = 8) *BRCA1* expression groups. Vaginal swabs were taken in duplicate for 16 S rRNA and metabolomics sequencing. Midwives or doctors collected cervical smear samples from participants using sterile swabs. The swab was rotated five times on the cervix, then placed in a PBS-filled freeze-storage tube and agitated ten times to release cells. Samples were stored at −80℃ for later analysis.

### 16 S rRNA Sequencing

Microbial genomic DNA was extracted from 14 vaginal swab samples using the DNeasy PowerSoil Pro Kit (TianGen, China). The DNA quality was checked with Nanodrop™ Eight (Thermo Fisher Scientific, USA) and 1.2% agarose gel electrophoresis (Invitrogen, USA). The V3-V4 region of the 16 S rRNA sequence was PCR-amplified from the purified DNA using Phusion^®^ Hot Start Flex 2×Master Mix (M0536S, NEB, USA). The forward primer used was 341 F (5’-CTACGGGNGGCWGCAG-3’) and the reverse primer was 805R (5’-GACTACHVGGGTATCTAATCC-3’). Library construction utilized the Next^®^ Ultra DNA Library Prep Kit (#E7645L, NEB). 16 S amplicon sequencing was performed on the Illumina NovaSeq 6000 platform by Shanghai ApexBio Biotechnology Co., LTD.

DADA2 was used for sequence quality filtering, trimming, chimera removal, merging paired-end sequences, denoising, and generating Feature information, also known as Amplicon Sequence Variants (ASVs). Species annotation was conducted for each ASV sequence to obtain species information and species-based abundance distribution for constructing Rank-abundance curves to assess species abundance and evenness. To assess microbial community diversity, Alpha diversity indices (Chao1, Shannon, Simpson) were calculated with QIIME2. For analyzing community changes, Beta diversity was evaluated using Bray-Curtis, Jaccard, Unweighted UniFrac, and Weighted UniFrac distances, and visualized through Principal Coordinates Analysis (PCoA).

Differential abundant taxa were identified using LEfSe, which applied the Kruskal-Wallis test to detect significant species abundance differences between groups. Linear Discriminant Analysis (LDA) was then used to estimate the effect size, with a Log (LDA) score cutoff of 2 indicating significant inter-group differences. A significance level of *p* < 0.05 indicated biological statistical significance. Microbial functions were predicted using the KEGG database.

### Liquid Chromatography-Mass spectrometry (LC-MS) analysis

Metabolites were extracted from vaginal swabs by adding methanol and 2-Chloro-L-phenylalanine, vortexing for 35 s, and sonicating in an ice-water bath for 5 min. The mixture was then kept at −20℃ for two hours, centrifuged at 4℃ and 1300 rpm for 15 min, and the supernatant was collected for LC-MS analysis.

The LC analysis was performed using a UHPLC system (ExionLCTM 100, SCIEX, USA). A T3 column (100 × 2.1 mm, 1.7 μm, 186009461, Waters, USA) was utilized under the following chromatographic conditions. In positive ion mode, the mobile phase consisted of 0.1% formic acid aqueous solution (A, 67-56-1, CNW, China) and acetonitrile (B, 75-05-8, CNW). In negative ion mode, the mobile phase comprised a 5 mM ammonium acetate aqueous solution with a pH of 9.0 (A) and acetonitrile (B). The parameters of the mobile phase are shown in Table 1. The column temperature was maintained at 45℃, and the injection volume was 1 µL.

Mass spectrometry was conducted using high-performance liquid chromatography (1290, Agilent, Germany) coupled with a Q Exactive Orbitrap mass spectrometer (IQLAAEGAAPFALGMAZR, Thermo Fisher Scientific). Mass scanning was performed in the range of 70 to 1000 m/z, with a primary resolution set at 70,000 and a secondary resolution of 17,500. Collision energies of 20, 40, and 60 eV were applied. The ion source parameters included a sheath gas flow rate of 40, auxiliary gas flow rate of 10, electrospray ionization source operating in positive or negative ion modes with a voltage of 3800 V, ion transfer tube temperature of 320 °C, and auxiliary gas heater temperature of 350℃.

The raw data were processed using ProteoWizard and XCMS programs, and substance identification was performed using the Compound Discover (OPTON-31061, Thermo Fisher Scientific) and OSI-SMMS (V1.0, ChemDataSolution, China) software in conjunction with the mzCloud database (https://www.mzcloud.org/). Multivariate statistical analysis, including Principal Component Analysis (PCA), Partial Least Squares Discriminant Analysis (PLS-DA), and Orthogonal Partial Least Squares Discriminant Analysis (OPLS-DA). Combining OPLS-DA with Student’s *t*-test was used to select differential metabolites. Metabolites with a Variable Importance for the Projection (VIP) ≥ 1 and *p* < 0.05 were considered significantly different. Finally, differential metabolites were analyzed for enriched pathways using the KEGG database.

**Table 1:** Mobile phase parameters table

Time (min)	Row (µL/min)	A (%)	B (%)
0	500	99	1
1.0	500	99	1
8.0	500	1	99
10.0	500	1	99
10.1	500	99	1
12.0	500	99	1

### Analysis of vaginal microbiota and metabolite correlation

A statistically significant interaction network between differential vaginal microbiota and metabolites was constructed using Cytoscape (V3.10.2, Scripps Research, USA). Spearman correlation analysis and heatmap visualization were employed to evaluate the associations between the composition of the vaginal microbial community and metabolite levels. Significant correlations were identified based on the criteria of |R| >0.6 and *p* < 0.05, leading to the identification of differentially associated microbiota and metabolites.

### Molecular docking of metabolites with *METTL1*

Docking studies of compounds, including 5-formamido-4-imidazolecarboxamide, cobalt-precorrin-7, inosine, L-lysine, and uridine, with *METTL1*, were performed using AutoDock VINA software (v1.1.2, NIH, USA). Compounds exhibiting binding energies ≤ −5.0 kJ/mol were identified as key molecules with significant affinity for the *METTL1* protein. Subsequent visualization and analysis of the compound-*METTL1* interactions were conducted using PYMOL software (v3.0.3, DeLano, USA). The three-dimensional structures of the compounds were sourced from the PubChem database (https://pubchem.ncbi.nlm.nih.gov/), whereas the three-dimensional structure of the *METTL1* protein was obtained from the RCSB Protein Data Bank (https://www.rcsb.org/).

### Cell culture and treatment

Normal human ovarian surface epithelial cells (IOSE-80, AW-CNH242, Abiowell, China) and HGSOC cell lines OVCAR3 (AW-CCH113, Abiowell), OVCAR5 (tings-1618165, Wanwu, China), and OVCAR8 (CL-0759, Procell, China) were cultured in RPMI-1640 medium supplemented with 10% FBS and 1% penicillin-streptomycin. All cells were incubated at 37℃ in a 5% CO_2_ humidified atmosphere. The expression of *METTL1* and *BRCA1* was evaluated using qRT-PCR and WB, and the HGSOC cell line with the most significant difference in *METTL1* expression was selected for functional studies.

*METTL1* overexpression (oe-METTL1, HG-HO015935. HonorGene, China) or knockdown plasmids (si-METTL1, HG-SH015935, HonorGene) and *BRCA1* overexpression plasmid (oe-BRCA1, HG-HO004656, HonorGene) were transfected into OVCAR8 cells using Lipofectamine 2000 reagent (11668-019, Invitrogen), and transfection efficiency was evaluated by qRT-PCR and WB. si-METTL1#1: GAGGAAAGAAAGCTTTCGAGTGG. si-METTL1#2: GCCTGAAGATTAGGGAAAATAAA. si-NC: TTACGGTACGTACGGTACGTAAC.

To elucidate the functions and underlying mechanisms of *METTL1* and *BRCA1* in HGSOC, OVCAR8 cells were subjected to the following group treatments: 1). si-NC group: cells transfected with si-NC. 2). si-METTL1 group: cells transfected with si-METTL1. 3). si-METTL1 + oe-NC group: cells transfected with si-METTL1 and oe-NC. 4). si-METTL1 + oe-BRCA1 group: cells transfected with si-METTL1 and oe-BRCA1.

OVCAR8 cells were exposed to varying concentrations (0, 0.1, 0.2, 0.4, 0.8, 1.6, 3.2 µM) of 5-formamidoimidazole-4-carboxamide ribotide (FAICAR, T63420, Yuanye, China). The optimal concentration for treatment was determined utilizing the Cell Counting Kit-8 (CCK-8) assay. To elucidate the role of FAICAR in high-grade serous ovarian cancer (HGSOC), OVCAR8 cells were divided into the following experimental groups: (Senkowski et al. 2023) Control group: cells maintained under standard culture conditions; (Janíková et al. 2021) FAICAR group: cells treated with 0.8 µM FAICAR for 24 h.

To explore the connection between FAICAR and the *METTL1/BRCA1* axis in HGSOC, OVCAR8 cells were subjected to the following group treatments: 1). Control: cells cultured normally. 2). FAICAR + oe-NC group: cells transfected with oe-NC and treated with 0.8 µM FAICAR for 24 h. 3). FAICAR + oe-METTL1 group: cells transfected with oe-METTL1 and treated with 0.8 µM FAICAR for 24 h. 4). FAICAR + oe-BRCA1 group: cells transfected with oe-BRCA1 and treated with 0.8 µM FAICAR for 24 h.

### WB

Proteins were extracted from cellular or homogenized tissue samples utilizing RIPA lysis buffer (P0013B, Biyuntian, China). The protein concentration was subsequently quantified using a BCA protein assay kit (Solarbio, PC0020). The resulting protein lysate was combined with loading buffer and subjected to boiling for 10 min. Total proteins were then resolved by electrophoresis on a 10% SDS-PAGE gel and transferred onto a nitrocellulose membrane. The membrane was blocked with 5% skim milk powder (AWB0004, Abiowell) and then incubated with the primary antibodies (Table 2) overnight at 4℃ on a shaker. Subsequently, the membrane was incubated with the secondary antibody, followed by the addition of ECL Plus hypersensitive luminescent solution (AWB0005, Abiowell). The membrane was imaged using a gel imaging system, and the exposed image strips were analyzed using Quantity One Professional grayscale analysis software.

**Table 2:** Primary antibody information

Name	Cat. number	MW (kDa)	Dilution rate	Company	Country
METTL1	14994-1-AP	30	1:1000	Proteintech	USA
BRCA1	83390-6-RR	300	1:20000	Proteintech	USA
m7G	ab286165	37	1:1000	Abcam	UK
U6	AWA57528	10	1:1000	Abiowell	China
GAPDH	#2118	37	1:5000	CST	USA

### Real-time quantitative PCR (RT-qPCR)

Total RNA was extracted from cell or homogenized tissue samples using TRIZOL (15596026, Thermo Fisher Scientific). The extracted RNA was reverse-transcribed into cDNA utilizing the HiFiScript cDNA Synthesis Kit (CW2569, CWBio, China). The PikoReal five-channel real-time PCR system (Thermo Fisher Scientific) along with UltraSYBR Mixture (CW2601, CWBio) were employed for conducting fluorescence quantitative PCR reactions. Primers utilized in this study were synthesized by Beijing Qingke Biotechnology Co., LTD., and their sequences can be found in Table 3. Data analysis was performed using the 2^-ΔΔCt method and normalized to GAPDH expression levels.

**Table 3:** qRT-PCR primer sequence

Primer	Sequences (5’−3’)	Product length
*METTL1*-F	GACCCACATTTCAAGCGGACA	86
*METTL1*-R	CCAACTCTTAGCACGTAGGCAT	
*BRCA1*-F	CCCCAGAAGAATTTATGCTCGT	167
*BRCA1*-R	TACCCATTTTCCTCCCGCAAT	
*GAPDH*-F	ACAGCCTCAAGATCATCAGC	104
*GAPDH*-R	GGTCATGAGTCCTTCCACGAT	

### CCK-8

To evaluate cell proliferation, 10 µL/well CCK-8 solutions were added to the cells cultured in 96-well plates and incubated at 37℃ for 4 h. The optical density values of the well at 450 nm were measured with a microplate reader (MB580, HEALES, China).

### 5-Ethynyl-2’-deoxyuridine (EdU) staining

The cell proliferation rate was assessed using the EdU Labeling Kit (C10310, RIB BIO, China). Cells were seeded into 96-well plates (1.5 × 10^4^ cells/well), treated as per experimental requirements, and incubated with 50 µM EdU medium (1:1000 ratio of reagent A to complete cell medium). After 2 h, the medium was removed, and cells were washed with PBS. Fixative solution containing 4% paraformaldehyde (N1012, NCM, China) was added and incubated at room temperature for 30 min. The fixative was discarded, and 50 µL of 2 mg/mL glycine solution was added, followed by a 5-minute incubation on a decolorizing shaker before discarding the glycine solution. After another PBS wash, 100 µL of 0.5% Triton X-100 was added and incubated for 10 min. Subsequently, 100 µL of 1× Apollo^®^ dye reaction solution (prepared from reagent B-E according to the kit instructions) was added and incubated at room temperature in the dark. After 30 min, cells were treated with 0.5% Triton X-100 for 10 min. Finally, 1 × Hoechst 33,342 staining solution (reagent F diluted with deionized water at a 100:1 ratio) was added and incubated at room temperature in the dark for 30 min. Live cells were visualized under a fluorescence microscope (Olympus, Tokyo, Japan).

### Flow cytometry

An apoptosis detection kit (KGA1105, KeyGen, China) was used to determine the degree of apoptosis. Simply put, a Binding Buffer of 500 µL was added to OVCAR8 cells in each group, and the binding buffer was gently blown into a single-cell suspension. Then 5 µL Annexin V-APC and 5 µL Propidium Iodide were added to each group, and the mixture was reacted at room temperature for 10 min without light. Observation and detection were performed within 1 h using flow cytometry (A00-1–1102, Beckman, USA).

### Transwell

The cells were suspended in a serum-free medium, and their density was adjusted to 2 × 10^6^ cells/mL. Cells (100 µL/well) were inoculated into Transwell chambers (3428, Corning) with or without Matrigel matrix gel, and then 500 µL medium containing 10% FBS was added to the lower chamber. After 48 h of culture, the chambers were removed. The cells were fixed with 4% paraformaldehyde. The cells were stained with 0.1% crystal violet dye (AWC0333a, Abiowell). The upper and outer surface cells are observed under a microscope, and images are taken.

### RNA Immunoprecipitation (RIP)-PCR

RIP-PCR was performed to investigate the interaction between METTL1 protein and BRCA1 mRNA using the Imprint^®^ RNA Immunoprecipitation kit (RIP, Sigma). Cells were lysed in an RIP lysis buffer on ice. Subsequently, 5 µg of METTL1 antibodies (14994-1-AP, Proteintech) or IgG antibody (B900620, Proteintech) were incubated with the protein A/G magnetic beads at room temperature for 30 min. The antibody complex and cell lysate were then incubated overnight at 4℃ to form the immunoprecipitation complex. Following this, the complex was treated with protease K at 55℃ for 30 min to extract the RNA bound to the METTL1 protein. The extracted RNA was reverse-transcribed into cDNA, and the level of *BRCA1* mRNA was quantified using RT-qPCR.

### RNA pull-down

Cells were lysed using RIPA lysis buffer (P0013B, Beyotime). At room temperature, a 50 pmol biotinylated *BRCA1* probe (RiboBio, China) was incubated with streptavidin-coated agarose magnetic beads (20353, Thermo Fisher Scientific) in an RNA capture buffer for 30 min. The bead-RNA mixture was then combined with 100 µL of cell lysate and rotated at 4℃ overnight. A portion of the lysate was saved at −20℃ for subsequent input analysis. The incubated bead-RNA-protein mixture was centrifuged at low speed, washed twice with 500 µL of Wash Buffer, and then eluted by adding 50 µL of Elution Buffer and shaking at 37℃ for 30 min. After centrifugation, 50 µL of the supernatant was collected, mixed with 12.5 µL of 5 × loading buffer, and heated in a water bath for 10 min. The abundance of METTL1 protein was identified by WB.

### In vivo tumor formation

Female athymic nude mice (6–7 weeks old) were obtained from Hunan Slake Jingda Experimental Animal Co., Ltd (China). The mice were subcutaneously injected with stably transfected or non-transfected OVCAR8 cells (200 µL, 8 × 10^6^ cells). Specifically: si-NC group: subcutaneous injection of OVCAR8 stably transfected with si-NC; si-METTL1 group: subcutaneous injection of OVCAR8 stably transfected with si-METTL1; si-METTL1 + oe-NC group: subcutaneous injection of OVCAR8 stably transfected with si-METTL1 + oe-NC; si-METTL1 + oe-BRCA1 group: subcutaneous injection of OVCAR8 stably transfected with si-METTL1 + oe-BRCA1. FAICAR group: subcutaneous injection of non-transfected OVCAR8 cells and 100 µL FAICAR (0.073 mg/L). Control group: subcutaneous injection of non-transfected OVCAR8 cells and an equal volume of saline. Subcutaneous tumor growth was monitored weekly, and on day 28, the mice were euthanized, and the subcutaneous tumors were harvested.

Nude mice were xenografted with stable transfected or untransfected OVCAR8 cells (200 µL, 8 × 10^6^ cells) and injected with 100 µL FAICAR (0.073 mg/L). Specifically, the FAICAR group: peritoneal injection of untransfected OVCAR8 and FAICAR; the Control group: peritoneal injection of untransfected OVCAR8 and equal amounts of normal saline. FAICAR + oe-METTL1 group: Peritoneal injection stably transfected OVCAR8 with oe-METTL1 and an equal amount of FAICAR. FAICAR + oe-BRCA1 group: peritoneal injection stably transfected OVCAR8 with oe-BRCA1 and an equal amount of FAICAR. Animal weight was tracked weekly, and on day 28, the mice were euthanized and obtained abdominal tumors from them.

### Data analysis

Data analysis was performed using GraphPad Prism 8.0. Differences between the two groups were analyzed using Student’s *t*-test or Wilcoxon rank-sum test. For more than two groups, statistical analyses were conducted using one or two-way analysis of variance and followed by Tukey’s post hoc test. Spearman correlation coefficient analysis was used to calculate the significance of the relationship between changed vaginal microbiota and differential metabolites, with *p* < 0.05 indicating statistically significant differences.

## Results

### *METTL1* and *BRCA1* were highly expressed in HGSOC

**Fig. 1:**
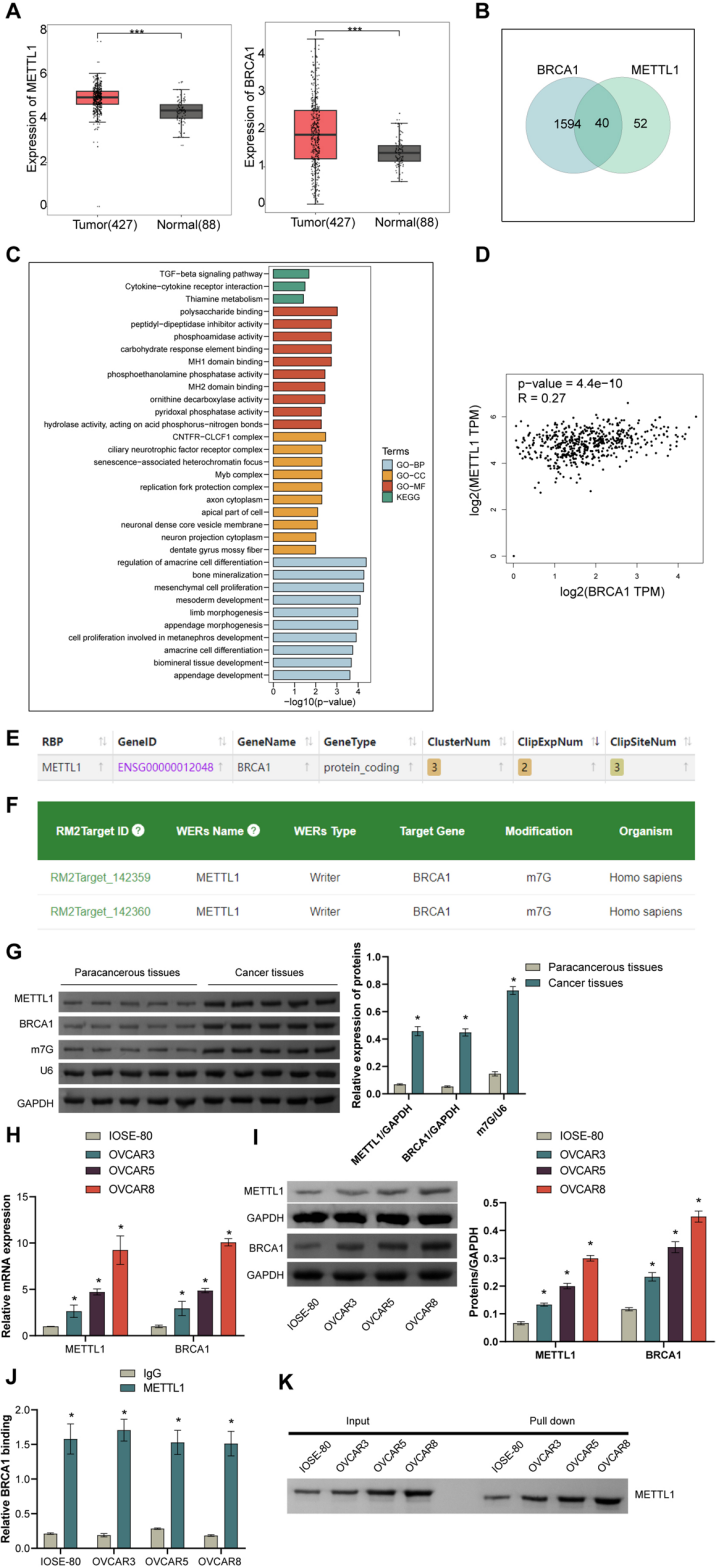
*METTL1* and *BRCA1* were highly expressed in HGSOC. **A** The expression of *METTL1* and *BRCA1* in OV was analyzed by TCGA. **B** Venn diagram shows the intersection of genes with high expression of both *METTL1* and BRCA1. **C** KEGG was used to predict the function of *METTL1* and *BRCA1* co-highly expressed genes in OV. **D** The correlation between *METTL1* and *BRCA1* was analyzed through the GEPIA database. **E** The interaction between *METTL1* and *BRCA1* was analyzed through CLIP-seq Data online website. **F** The combination of *METTL1* and *BRCA1* was predicted through the RMATarget database. **G** The expression of METTL1, BRCA1 and m7G in tumors and paired para-cancerous tissues was detected by WB. **H** The RNA expression of *METTL1* and *BRCA1* in IOSE-80 and HGSOC cells (OVCAR3, OVCAR5, OVCAR8) was detected by qRT-PCR. **I** The protein level of METTL1 and BRCA1 in IOSE-80 and HGSOC cells (OVCAR3, OVCAR5, OVCAR8) was detected by WB **J**-**K**. The binding regulatory relationship of METTL1 protein to *BRCA1* mRNA in the HGSOC cell line was detected by RIP-PCR and RNA pull-down. *p < 0.05 compared with paracancer tissue, IOSE-80 cells or IgG group. The comparison between the two groups was performed using the *t*-test, and the comparison between the multiple groups was performed using ANOVA. Cell assay (**G**-**K**) with 3 biological replicates and 3 technical replicates

TCGA analysis based on TCGA and GTEx data showed that *METTL1* and *BRCA1* were highly expressed in OV (Fig. 1A). The differential genes that are highly expressed with *METTL1* and *BRCA1* in OV were intersected, as shown in Venn Fig. 1B, and a total of 40 intersection up-regulated genes were obtained. Functional analysis by KEGG showed that the genes with high expression of *METTL1* and *BRCA1* play an important role in polysaccharide-binding, regulation of cell differentiation, CNTFR-CLCF1 and peptidyl-dipeptidase inhibitor activity were enriched (Fig. 1C).

Analysis based on the GEPIA database showed that *METTL1* was positively correlated with *BRCA1* (Fig. 1D). Based on the CLIP-seq Data, analysis shows that METTL1 might interact with *BRCA1* (Fig. 1E). Based on the m7GHub database to predict found that *BRCA1* mRNA has several potential m7G modification sites (Supplementary Table 1). Based on the RMATarget database (http://rm2target.canceromics.org/#/home), it predicted that *METTL1* may be as *BRCA1* m7G Writer (Fig. 1F).

Next, we examined the expression of *METTL1*, BRCA1, and m7G. WB results showed that the levels of METTL1 (*p* < 0.0001), BRCA1 (*p* < 0.0001) and m7G (*p* < 0.0001) in cancer tissues were significantly higher than those in adjacent tissues (Fig. 1G, all *p* < 0.0001). A qRT-PCR and WB detection results showed that compared with IOSE-80, the RNA expressions of *METTL1* and *BRCA1* and protein level of METTL1 and BRCA1 in OVCAR3 (*METTL1* RNA, *p* = 0.043. METTL1 Protein, *p* < 0.0001. *BRCA1* RNA, *p* = 0.0148. BRCA1 Protein, *p* < 0.0001), OVCAR5 (*METTL1* RNA, *p* < 0.0001. METTL1 Protein, *p* < 0.0001. *BRCA1* RNA, *p* < 0.0001. BRCA1 Protein, *p* < 0.0001). and OVCAR8 (*METTL1* RNA, *p* < 0.0001. METTL1 Protein, *p* < 0.0001. *BRCA1* RNA, *p* < 0.0001. BRCA1 Protein, *p* < 0.0001) cells were significantly increased (Fig. 1H and I), in which the high expression of OVCAR8 was the best. We selected OVCAR8 for follow-up experiments.

In addition, in view of the possibility of METTL1 binding to *BRCA1* predicted by the database, we confirmed the binding regulatory relationship between METTL1 and *BRCA1* through RIP-PCR and RNA pull-down experiments (Fig. 1J (all *p* < 0.0001) and Fig. 1K). These results suggest that *METTL1* may be dependent on m7G modification to promote high expression of *BRCA1* in HGSOC.

### *METTL1*-mediated m7G modification regulates the expression of *BRCA1* in HGSOC cell

**Fig. 2:**
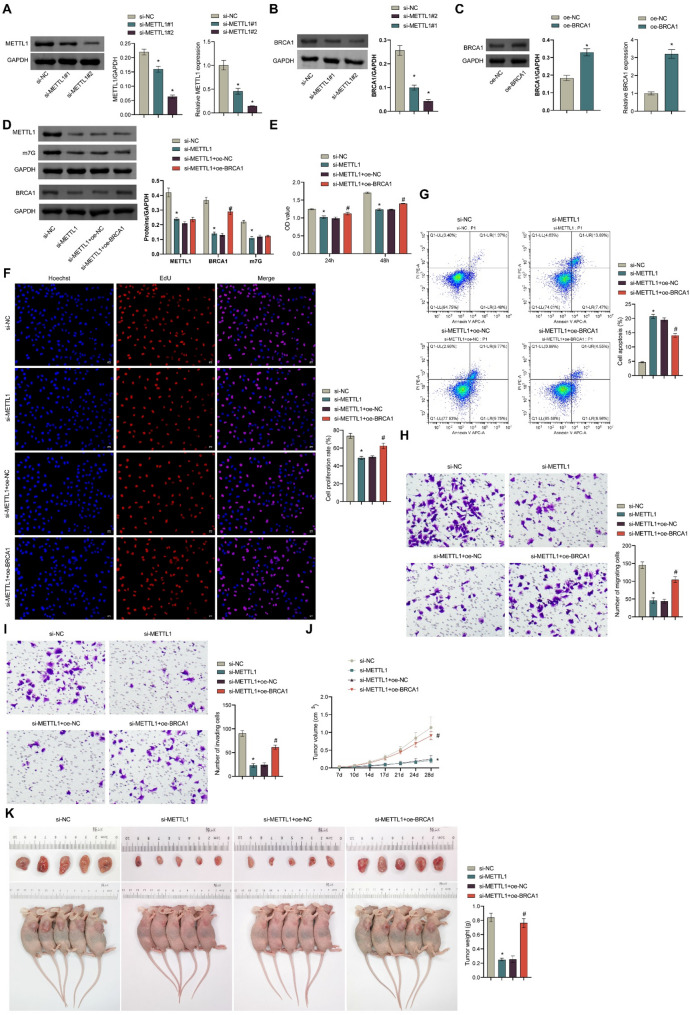
*METTL1*-mediated m7G modification regulates BRCA1 expression in HGSOC cells. **A** The interference efficiency of si-METTL1 was detected by qRT-PCR and WB. **B** The expression of the BRCA1 protein, which interferes with *METTL1* expression in OVCAR8 cells, was detected by WB. **C** The overexpression efficiency of oe-BRCA1 was detected by qRT-PCR and WB. **D** The expression of METTL1, m7G, and BRCA1 in OVCAR8 cells silenced by METTL1 or overexpressed by BRCA1 was detected by WB. **E** CCK8 was used to detect the changes in cell viability after *METTL1* silencing or *BRCA1* overexpression. **F** Cell proliferation after *METTL1* silencing or *BRCA1* overexpression was evaluated by EdU. **G** The apoptosis of cells after *METTL1* silencing or *BRCA1* overexpression was detected by flow cytometry. **H**-**I** Transwell assay was used to detect cell migration and invasion after *METTL1* silencing or *BRCA1* overexpression. A xenograft tumor model was constructed in nude mice by subcutaneous injection of stably transfected (si-NC, si-METTL, si-METTL + oe-BRCA1) or untransfected OVCAR8 cells. **J** Tumor volume change curve. K. Tumor body observation and tumor weight. *p < 0.05 compared with the si-NC group. #*p* < 0.05 compared with si-METTL1 + oe-NC group. The comparison between the two groups was performed using the *t*-test, and the comparison between the multiple groups was performed using ANOVA. Cell Assay (**A**-**I**) with 3 biological replicates and 3 technical replicates. Animal studies (**J**-**K**) with 5 biological replicates and 3 technical replicates

To investigate the functional roles of *METTL1* and *BRCA1* in HGSOC, we successfully constructed OVCAR8 cells with METTL1 knockdown (Fig. 2A, si-NC vs. si-METTL1#1, protein, *p* = 0.0004, mRNA, *p* = 0.0002. si-NC vs. si-METTL1#2, protein, *p* < 0.0001, mRNA, *p* < 0.0001) and found that BRCA1 protein levels in the cells were lower than in the si-NC group (Fig. 2B, si-NC vs. si-METTL1#1, *p* < 0.0001. si-NC vs. si-METTL1#2, *p* < 0.0001). Furthermore, we successfully overexpressed BRCA1 in OVCAR8 cells (Fig. 2C, protein, *p* = 0.0006. mRNA, *p* = 0.0041).

In the OVCAR8 cells, we conducted a transfection of si-METTL1 alone or in combination with oe-BRCA1. WB results revealed a notable reduction in METTL1, m7G, and BRCA1 levels in the si-METTL1 group compared to the si-NC group (all *p* < 0.0001). Furthermore, a notable increase in BRCA1 levels was observed in the si-METTL1 + oe-BRCA1 group (*p* < 0.0001), while the levels of METTL1 and m7G showed no significant difference in comparison to the si-METTL1 + oe-NC group. (Fig. 2D). The CCK-8 (si-NC vs. si-METTL1, *p* < 0.0001. si-METTL1 + oe-NC vs. si-METTL1 + oe-BRCA1, *p* < 0.0001) and EdU assays (si-NC vs. si-METTL1, *p* < 0.0001. si-METTL1 + oe-NC vs. si-METTL1 + oe-BRCA1, *p* = 0.0009) demonstrated that the knockdown of *METTL1* expression markedly suppressed the proliferation of OVCAR8 cells (Fig. 2E and F). Flow cytometry and Transwell experiments demonstrated that interference with *METTL1* expression markedly inhibited the migration and invasion capabilities of OVCAR8 cells (Fig. 2H, all *p* < 0.0001) and promoted apoptosis (Fig. 2G, all *p* < 0.0001). The overexpression of *BRCA1* was observed to significantly reverse the suppressed proliferation, migration, and invasion behaviors of OVCAR8 induced by *METTL1* downregulation, while also inhibiting apoptosis (Fig. 2E and I).

Subsequently, in vivo studies on subcutaneous tumor xenografts in nude mice demonstrated that *METTL1* knockdown could inhibit tumor growth, while overexpression of *BRCA1* could reverse the inhibited tumor growth caused by *METTL1* downregulation (Fig. 2J and K, all *p* < 0.0001). These findings suggest that *METTL1* may influence the progression of HGSOC by regulating the expression of *BRCA1* through m7G modification.

### 16 S rRNA sequencing analysis confirmed *Lactobacillus* was enriched in the BRCA1 high-expression group

**Fig. 3:**
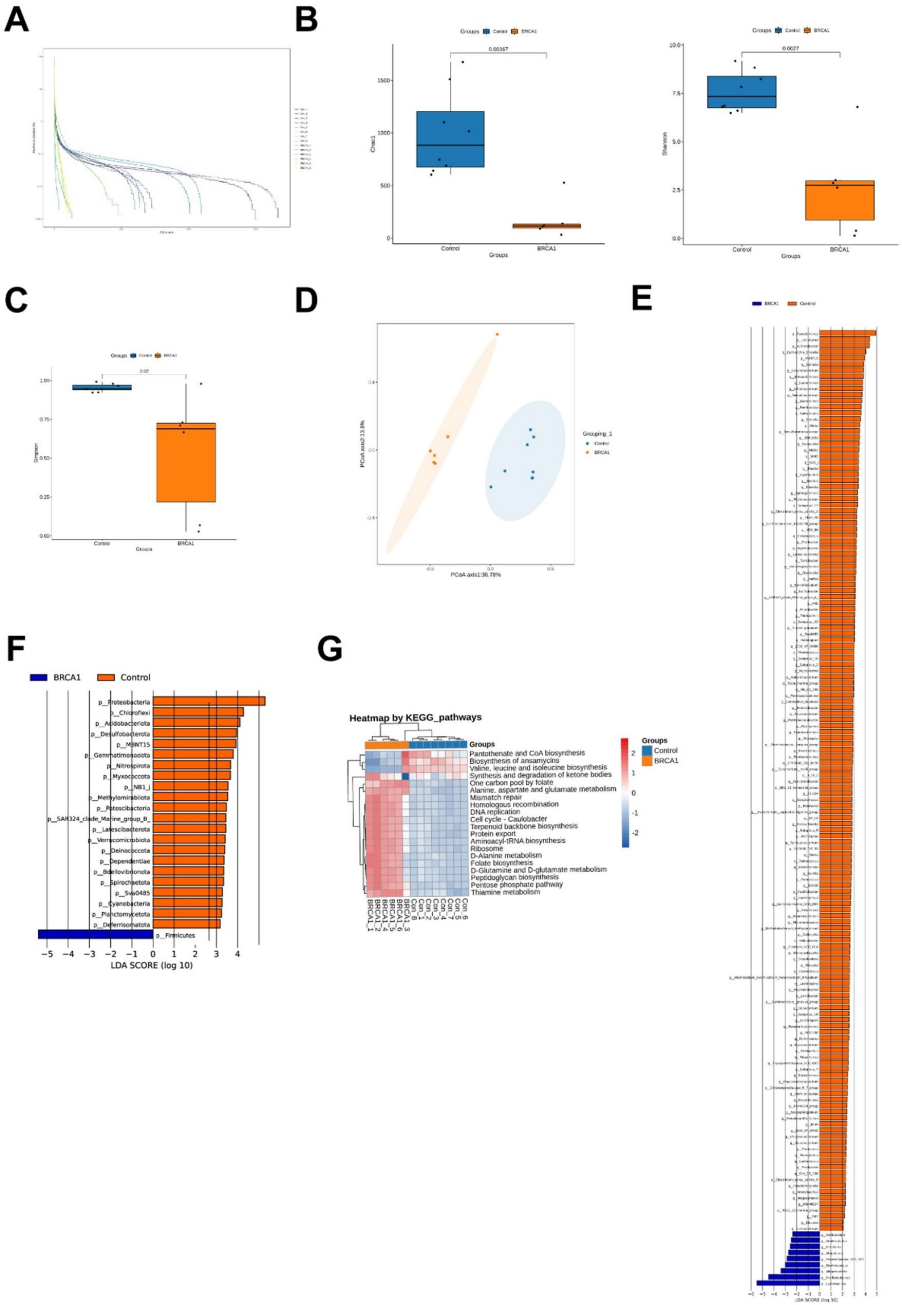
*Lactobacillus* was enriched in the *BRCA1* high-expression group. **A** Species abundance and evenness were evaluated by the Rank abundance curve. **B**-**C** Alpha diversity analysis was used to assess the richness and diversity of microbial communities. **D** beta diversity analysis was used to assess differences in species abundance distribution between groups. **E**-**F** The analysis of the relative abundance of vaginal microbiota species at phylum and genus levels. **G** KEGG was used to analyze functional changes associated with microbiota dysregulation. Control group, *N* = 8. BRCA1 group, *N* = 6. LEfSe was used to identify taxa with differential abundance, and Kruskal-Wallis test was used to detect significant differences in species abundance between groups. Log (LDA) 2, *p* < 0.05 was considered significant difference

To investigate whether there is a correlation between *BRCA1* expression and vaginal microbiota, we divided HGSOC patients into two groups based on *BRCA1* expression levels: *BRCA1* high expression (referred to as *BRCA1* group) and *BRCA1* low expression (referred to as Control group) and conducted 16 S rRNA sequencing analysis on their vaginal samples. The Rank abundance curve in Fig. 3A indicates a significantly lower richness of vaginal microbiota species in the *BRCA1* group compared to the Control group. Subsequent analysis of alpha-diversity indices showed a significant decrease in the Chao1 index (*p* < 0.00067), Shannon index (*p* < 0.0027), and Simpson index (*p* < 0.02) in the *BRCA1* group compared to the Control group, as shown in Fig. 3B and C.

Beta-diversity analysis conducted through PCoA revealed significant differences in the community composition of vaginal microbiota between HGSOC patients with high and low *BRCA1* expression, as depicted in Fig. 3D. Distribution analysis at the phylum and genus levels of cervicovaginal microbiota showed a notable increase in the abundance of *Firmicutes* and *Lactobacillus* in HGSOC patients with high *BRCA1* expression, as shown in Fig. 3E (Log (LDA) 2, *p* < 0.05) and Fig. 3F (Log (LDA) 2, *p* < 0.05).

Furthermore, we performed KEGG analysis to investigate functional changes related to cervicovaginal microbiota dysbiosis in HGSOC patients. KEGG enrichment analysis indicated significantly weakened metabolic pathways such as Pantothenate and CoA biosynthesis, and Valine, leucine, and isoleucine biosynthesis in HGSOC with high *BRCA1* expression. They were primarily enriched in D-glutamine and D-glutamate metabolism, D-Alanine metabolism, Pentose phosphate pathway, and Thiamine metabolism, as shown in Fig. 3G. These results suggest that the progression of HGSOC is associated with a decrease in cervicovaginal microbiota diversity. Specifically, in the high *BRCA1* expression group, the abundance changes of the genus *Lactobacillus*, belonging to the phylum *Firmicutes*, were notably significant, possibly linked to the differential expression of *BRCA1*.

### Metabolomic sequencing analysis of the cervical-vaginal metabolites in HGSOC patients

**Fig. 4:**
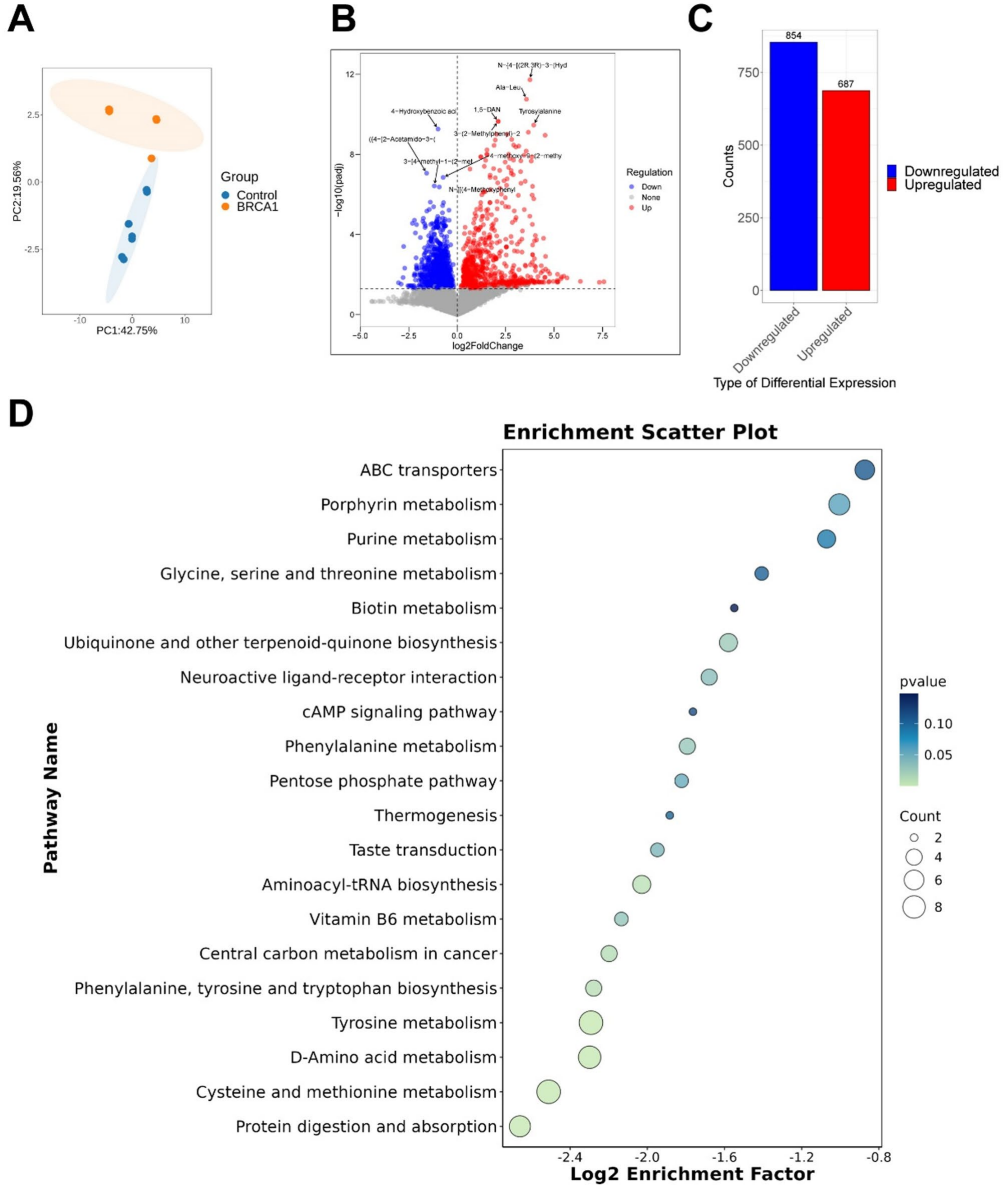
Metabolomic sequencing analysis of the cervical-vaginal metabolites. **A** PCA was used to analyze the variability between the *BRCA1* group and the Control group. **B** The significance of metabolite changes between the *BRCA1* group and the Control group was demonstrated by volcanic maps. **C** Quantitative statistics of differential metabolites between the *BRCA1* group and Control group. **D **The enrichment pathway of differential metabolites was analyzed by KEGG. Control group, *N* = 8. BRCA1 group, *N* = 6. OPLS-DA combined with Student ‘ST test was used to select differential metabolites. VIP ≥ 1 and *p* < 0.05 are considered to have significant differences

To further investigate the potential correlation between *BRCA1* expression and metabolites in the cervicovaginal microbiota, we conducted untargeted metabolomic profiling of cervical-vaginal swab samples using LC-MS. PCA analysis revealed significant differences in metabolites present in the cervical-vaginal swab samples between patients with high and low *BRCA1* expression (Fig. 4A). The volcano plot in Fig. 4B illustrates these differences (logFC > log2(1.5) & *p* < 0.05). Specifically, there were 854 significantly up-regulated and 687 significantly downregulated differential metabolites in the *BRCA1* high-expression cohort (Fig. 4C).

Subsequently, we performed KEGG analysis on these differential metabolites to elucidate the functional changes associated with inter-group differential metabolites. The bubble plot in Fig. 4D demonstrates significant enrichment of pathways such as Protein digestion and absorption, Cysteine and methionine metabolism, D-Amino acid metabolism, Tyrosine metabolism, and Central carbon metabolism in cancer in HGSOC patients with high *BRCA1* expression (Fig. 4D). These pathways may represent metabolic preference in the progression of HGSOC. These findings suggest that high *BRCA1* expression may lead to significant changes in endogenous physiological metabolites by disrupting vaginal metabolism, thereby promoting tumorigenesis.

### Microbiota-metabolite interaction analysis

**Fig. 5:**
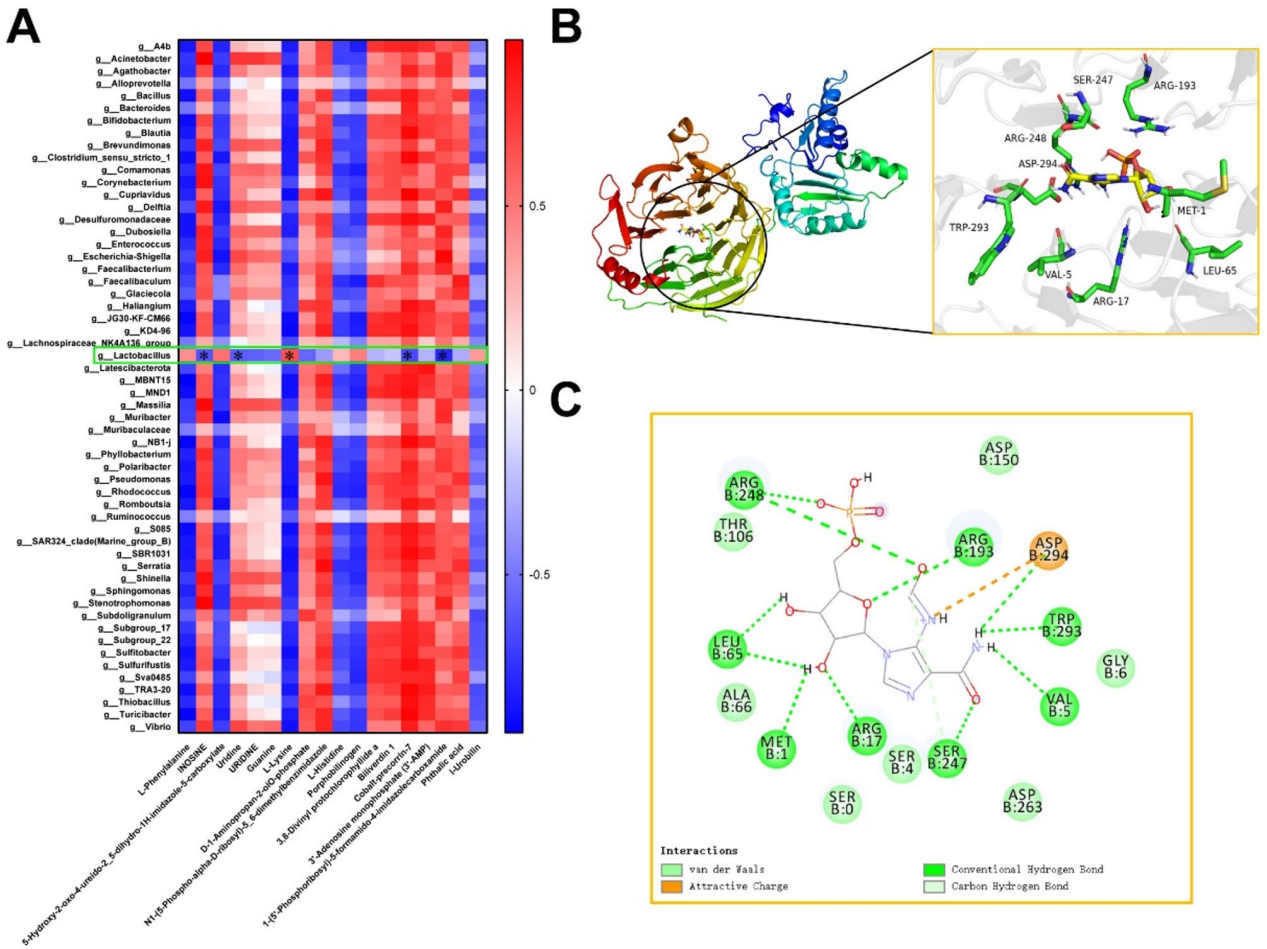
Microbiota-metabolite interaction analysis. **A** Heatmap of correlation analysis between microorganisms and metabolites. **B**-**C** Molecular docking analysis of 5-formamidoimidazole-4-carboxamide ribotide and *Lactobacillus*. Spearman correlation analysis was used to evaluate the correlation between vaginal microbiota composition and metabolite levels *Indicates the correlation coefficient |R| >0.6

In an attempt to investigate the role of changes in metabolites resulting from the differential expression of *BRCA1*-induced microbiota-host interactions in the progression of HGSOC, we constructed a microbiota-metabolite interaction analysis using Spearman. As shown in the heatmap in Fig. 5A (|R| >0.6 and *p* < 0.05), the majority of metabolites exhibit a strong correlation with differential microbial communities. Based on the significant increase in the abundance of *Lactobacillus* in the high *BRCA1* expression cohort, we selected metabolites that showed notable correlation with *Lactobacillus* for further analysis. At the genus level, 5-formamidoimidazole-4-carboxamide ribotide (*R*=−0.785, *p* < 0.05), INOSINE (*R*=−0.622, *p* < 0.05), Cobalt-precorrin-7 (*R*=−0.662, *p* < 0.05), and Uridine (*R*=−0.600, *p* < 0.05) exhibited significant negative correlations with *Lactobacillus*, while L-Lysine (*R* = 0.657, *p* < 0.05) showed a significant positive correlation.

To investigate the potential roles of these metabolites in relation to the high expression of *BRCA1* mediated by *METTL1* in the progression of HGSOC, we performed molecular docking analysis of these metabolites that exhibited significant correlation with *Lactobacillus* with *METTL1*. Specifically, the binding energy between 5-formamidoimidazole-4-carboxamide ribotide and METTL1 was calculated to be −7.5 kcal/mol (Fig. 5B and C). These results suggest that the increased abundance of *Lactobacillus* may lead to a decrease in the level of 5-formamidoimidazole-4-carboxamide ribotide in the vaginal environment of HGSOC patients, which could be linked to the high expression of *BRCA1* mediated by *METTL1*.

### Metabolite 5-formamidoimidazole-4-carboxamide ribotide inhibits HGSOC progression in vitro and in vivo by regulating *METTL1/BRCA1*

**Fig. 6:**
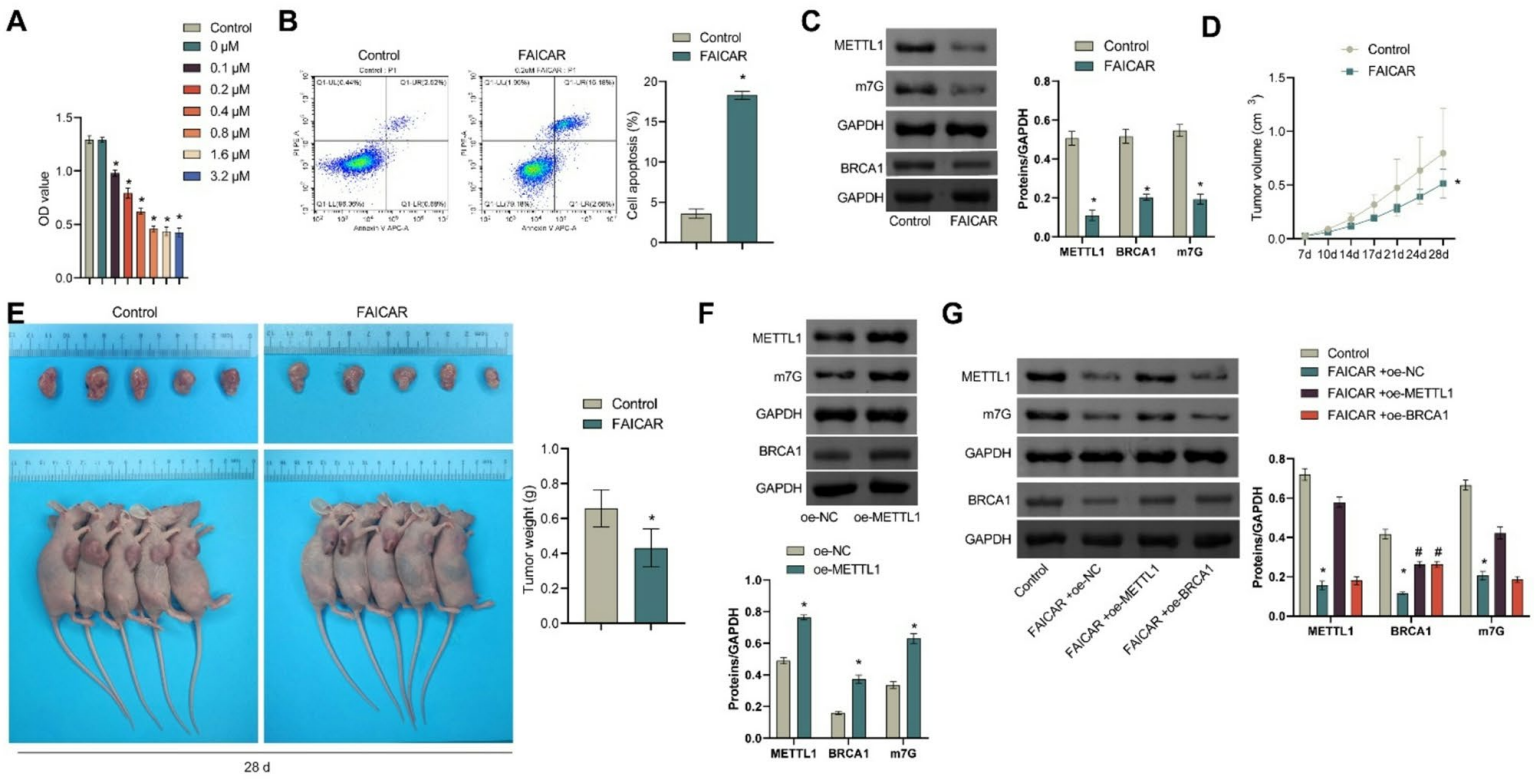
Metabolite 5-formamidoimidazole-4-carboxamide ribotide regulated *METTL1/BRCA1 *to inhibit HGSOC Development in vitro and in vivo. **A** The effects of different concentrations of 5-formamidoimidazole-4-carboxamide ribotide on cell viability were detected by CCK8. **B** The effect of 0.8 µM 5-formamidoimidazole-4-carboxamide ribotide on apoptosis was detected by flow cytometry. **C** WB was used to detect the effect of 0.8 µM 5-formamidoimidazole-4-carboxamide ribotide on the expression of *METTL1*, m7G and BRCA1. OVCAR8 cells and 5-formamidoimidazole-4-carboxamide ribotide were injected subcutaneously. **D** Tumor volume change curve. **E** Tumor map and tumor weight. **F** WB was used to detect the expression of *METTL1*, m7G and BRCA1 in OVCAR8 cells transfected with oe-METTL1. OVCAR8 was transfected with oe-METTL1 or oe-BRCA1 and treated with 5-formamidoimidazole-4-carboxamide ribotide. **G** The expressions of METTL1, m7G and BRCA1 in each group were detected by WB. **H** EdU was used to detect the proliferation ability of cells in each group. **I**-**J** Transwell was used to detect the migration and invasion ability of cells in each group. Stably overexpressed *METTL1* or *BRCA1* or untransfected OVCAR8 cells and 5-formamidoimidazole-4-carboxamide ribotide were injected into the peritoneum of nude mice. **K** Body weight change curve of nude mice. **L** Tumor map and tumor weight. *p < 0.05 compared with the Control group. #*p* < 0.05 compared with FAICAR group or FAICAR + oe-NC group. Note: FAICAR is 5-formamidoimidazole-4-carboxamide ribotide. The comparison between the two groups was performed using the *t*-test, and the comparison between the multiple groups was performed using ANOVA. Cell Assay (**A**-**C**, **F**-**J**) with 3 biological replicates and 3 technical replicates. Animal studies (**D**-**E**, **K**-**L**) with 5 biological replicates and 3 technical replicates

We treated OVCAR8 cells with different concentrations of 5-formamidoimidazole-4-carboxamide ribotide (0, 0.1, 0.2, 0.4, 0.8, 1.6, 3.2 µM) to investigate its function in HGSOC. CCK-8 results showed that 5-formamidoimidazole-4-carboxamide ribotide treatment significantly inhibited cell viability in a concentration-dependent manner compared to the control group (Fig. 6A, all *p* < 0.0001). Additionally, flow cytometry and WB analysis revealed that treatment with 0.8 µM of 5-formamidoimidazole-4-carboxamide ribotide significantly promoted cell apoptosis (Fig. 6B, *p* = 0.0016) and reduced the expression of METTL1, m7G, and BRCA1 (Fig. 6C, all *p* < 0.0001). Subcutaneous xenograft experiments in nude mice further confirmed that 5-formamidoimidazole-4-carboxamide ribotide treatment significantly inhibited tumor growth (Fig. 6D (28 d, *p* < 0.0001.) and Fig. 6E (*p* = 0.004)).

Subsequently, we successfully constructed OVCAR8 cells overexpressing METTL1, leading to a significant increase in BRCA1 and m7G levels in these cells (Fig. 6F, all *p* < 0.0001). We then transfected OVCAR8 cells with oe-METTL1, oe-BRCA1, or oe-NC and treated them with 5-formamidoimidazole-4-carboxamide ribotide. qRT-PCR and WB analysis revealed that compared to the control group, the expression of METTL1, m7G, and BRCA1 was significantly decreased in the FAICAR + oe-NC group (all *p* < 0.0001). However, in the FAICAR + oe-METTL1 group, the expression of METTL1, m7G, and BRCA1 was significantly increased compared to the FAICAR + oe-NC group (all *p* < 0.0001). In the FAICAR + oe-BRCA1 group, BRCA1 expression was significantly increased compared to the FAICAR + oe-NC group (*p* < 0.0001), while METTL1 and m7G showed no significant changes (Fig. 6G).

Results from EdU and Transwell assays showed that 5-formamidoimidazole-4-carboxamide ribotide treatment significantly inhibited cell proliferation, migration and invasion compared to the control group (all *p* < 0.0001). However, overexpression of *METTL1* or *BRCA1* abrogated the inhibitory effect of 5-formamidoimidazole-4-carboxamide ribotide on the proliferation (FAICAR + oe-NC vs. FAICAR + oe-METTL1, *p* = 0.0006. FAICAR + oe-NC vs. FAICAR + oe-BRCA1, *p* = 0.0012), migration (FAICAR + oe-NC vs. FAICAR + oe-METTL1, *p* = 0.0008. FAICAR + oe-NC vs. FAICAR + oe-BRCA1, *p* = 0.0014) and invasion (FAICAR + oe-NC vs. FAICAR + oe-METTL1, *p* = 0.0168. FAICAR + oe-NC vs. FAICAR + oe-BRCA1, *p* = 0.0282) of OVCAR8 cells (Fig. 6H and J).

Additionally, we injected HGSOC cells stably transfected with *METTL1* or *BRCA1* overexpression vectors or oe-NC and 5-formamidoimidazole-4-carboxamide ribotide into the peritoneum of nude mice. Consistent with previous results, 5-formamidoimidazole-4-carboxamide ribotide treatment inhibited tumor growth. Importantly, overexpression of *METTL1* or *BRCA1* counteracted the anti-tumor effects of 5-formamidoimidazole-4-carboxamide ribotide (Fig. 6K (28 d, all *p* < 0.0001) and Fig. 6L (Control vs. FAICAR + oe-NC, *p* < 0.0001. FAICAR + oe-NC vs. FAICAR + oe-METTL1, *p* = 0.0206. FAICAR + oe-NC vs. FAICAR + oe-BRCA1, *p* = 0.0101)).

## Discussion

In this study, we discovered that *METTL1* boosts *BRCA1* expression through m7G modification, thereby facilitating cell proliferation and tumor growth in HGSOC. Furthermore, we identified associations between differences in *BRCA1* expression, vaginal microbiome dysregulation, and metabolic imbalances in patients with HGSOC. Specifically, our research found a significant increase in the abundance of *Lactobacillus* in the cohort with high *BRCA1* expression, which potentially led to decreased levels of 5-formamidoimidazole-4-carboxamide ribotide in the vaginas of HGSOC patients. The administration of the vaginal metabolite 5-formamidoimidazole-4-carboxamide ribotide to HGSOC cells and xenografted tumor models in nude mice resulted in the inhibition of cell proliferation and tumor growth of HGSOC, which was achieved by the inhibition of the *METTL1/BRCA1* axis.

Increasing evidence from various studies highlights the critical role of abnormal m7G modification in cancer pathogenesis (Zhao et al. 2024), which is involved in key biological processes such as tumor cell proliferation, angiogenesis and metastasis, and it is related to the immune regulation of the tumor microenvironment (Zhang et al. 2024; Han et al. 2024a, b). As a key methyltransferase involved in m7G modification, *METTL1* has been implicated in the methylation of m7G sites in multiple target genes, showing close associations with the progression and poor prognosis of diverse cancers such as glioblastoma (Wang et al. 2024), liver cancer (Dong et al. 2024), osteosarcoma (Wang et al. 2023), and castration-resistant prostate cancer (Zhang et al. 2023). Notably, individuals with *BRCA1* have an elevated risk of developing HGSOC (Tee et al. 2024). Our bioinformatics analysis and experimental validation uncovered high expressions of *METTL1* and *BRCA1* in HGSOC. Prior studies have demonstrated that the inflammatory microenvironment triggered by lipopolysaccharides drives the advancement of bladder cancer (Tang et al. 2024). The differentiation of myeloid inhibitory cells contributes to tumor progression and immune evasion (Guo et al. 2024; Dahal et al. 2024). CNTFR-CLCF1 signaling has been reported to favor the growth of lung cancer tumors (Kim et al. 2019). Through KEGG analysis, we identified 40 up-regulated genes that coincide with the high expressions of *METTL1* and *BRCA1*, playing crucial roles in polysaccharide binding, carbohydrate binding, regulation of cell differentiation, and CNTFR-CLCF1 signaling etc. These findings suggest involvement in polysaccharide metabolism, cell differentiation, and CNTFR-CLCF1 signaling pathways that may facilitate the malignant progression of HGSOC.

The functional investigation of *METTL1* and *BRCA1* revealed that *METTL1* mediated the upregulation of *BRCA1* through m7G modification, thereby promoting cell proliferation and tumor growth in HGSOC. Previous studies have shed light on the mechanisms underlying PARPi resistance in OC patients with HR repair defects, suggesting a potential link to the overexpression of *BRCA1* hypomorphs resulting from the splicing out of mutated exons from *BRCA1* splicing isomers ∆11 and ∆11q (Nesic et al. 2024). *BRCA1* mutations have been shown to diminish the effectiveness of anti-PD-L1 immunotherapy in OC tumors (Park et al. 2024). Research by Izabela L. et al. has indicated significantly higher expression of *BRCA1* in the cancer tissues of breast cancer patients with *BRCA1* mutations compared to *BRCA1* wild-type cancer tissues (Izabela et al. 2024). Additionally, investigations by Guffanti F. et al. have demonstrated the utility of quantifying *BRCA1* expression in predicting the therapeutic response to olaparib in OC xenotransplantation models (Guffanti et al. 2024). These findings suggest that targeting *METTL1* and *BRCA1* could hold promise as a therapeutic approach for HGSOC.

Recent studies have highlighted the significance of microbiota imbalance in the gut, tumor tissue, and the vaginal and cervical regions, as well as the associated changes in metabolites, in relation to the progression and treatment of OC (Nené et al. 2019; González-Sánchez and DeNicola 2021; Li et al. 2023). It has been reported that OC tissue samples exhibit significantly reduced microbiome diversity and richness, with a notable increase in the abundance of Proteobacteria at the phylum level and an elevated ratio of *Proteobacteria*/*Firmicutes* (Zhou et al. 2019). Furthermore, there is a tendency towards increased levels of *Firmicutes*, *Actinomycetes*, and *Proteobacteria* in the intestinal microbiome of OC and endometrial cancer patients (Mori et al. 2019). Through 16 S rRNA sequencing in our study, we discovered that the species diversity and richness of vaginal microbiota were lower in patients with high *BRCA1* expression compared to those with low *BRCA1* expression. Additionally, at the phylum level, an increase in *Firmicutes* abundance was observed in patients with high *BRCA1* expression. Discrepancies in these results may stem from sample variations, such as differences in anatomical locations. Moreover, OC comprises multiple histological subtypes with distinct pathogenesis and tumor microenvironments, possibly accounting for differences in vaginal microflora between HGSOC and other OC subtypes (Kurman and Shih Ie 2010).

At the genus level, we found that the relative abundance of *Lactobacillus* in the *BRCA1* high-expression cohort was elevated. This contrasts with previous findings suggesting that the vaginal microbiota of healthy women is predominantly composed of *Lactobacillus* species ((2012) 2012). However, other studies have indicated variations where *Lactobacillus* may not always dominate the vaginal microbiome in some healthy women, with groups like Pseudomonas, Atopola, and Gardendella being prevalent, possibly due to individual differences. The diversity of vaginal microbial composition, even in healthy women, is significantly influenced by factors such as host race, age, contraceptive method, and physiological cycle (Ravel et al. 2011; Donders et al. 2017; Chaban et al. 2014). Additionally, while *Lactobacillus* is generally considered beneficial in the gut, Hezaveh K found that indole produced by *Lactobacillus L. murinus* during tryptophan metabolism may promote the growth of pancreatic cancer (Hezaveh et al. 2022). Moreover, the accumulation of lactic acid is known to facilitate angiogenesis, cell migration, and metastasis of tumor cells, contributing to tumor progression (Pant et al. 2024; Kooshan et al. 2024). Based on our 16 S rRNA sequencing results, we hypothesize that the increased abundance of *Lactobacillus* may promote the high expression of *METTL1* and *BRCA1*, consequently advancing the progression of HGSOC.

Functional predictions derived from the KEGG database using 16 S rRNA data in HGSOC patients with high *BRCA1* expression suggested a significant weakening in metabolism related to Pantothenate and CoA biosynthesis and Valine, leucine, and isoleucine biosynthesis. Notably, they were predominantly enriched in D-Glutamine_and_D-glutamate_metabolism, D-Alanine_metabolism, Pentose phosphate pathway, and Thiamine metabolism. Recent studies have highlighted the critical role of the Pantothenate and CoA biosynthesis pathways in breast cancer progression (AlMalki et al. 2024), and it b has been reported to be related to glucose metabolism and tumor immunity (Liu et al. 2024; St Paul et al. 2021; Bourgin et al. 2022). Yi Y et al. demonstrated the disruption of Pantothenate and CoA biosynthesis pathways in colorectal cancer, possibly contributing to limited energy supply and the deficiency of anti-tumor effector T cells in these patients (Yi et al. 2023). Valine, leucine, and isoleucine biosynthesis have been associated with breast cancer recurrence (Yang et al. 2024a, b), with significant enrichment in patients with recurrent HGSOC compared to the primary disease (Reyes et al. 2019), and enhanced activity in this metabolic pathway has been shown to promote T-cell-mediated cytotoxicity of cancer cells (Yang et al. 2024a, b). These findings suggest that alterations in metabolic pathways influenced by dysregulated vaginal flora associated with BRCA1 expression may compromise the host’s anti-tumor immune response, thereby impacting the progression of HGSOC.

Studies have revealed the involvement of bacterial metabolite signaling, such as the up-regulation of lipopolysaccharides and lysophospholipids and the down-regulation of tryptophan metabolites, in the pathogenesis of OC (Sipos et al. 2021). However, some bacterial metabolites have shown beneficial effects, with short-chain fatty acids and bile acids inhibiting cancer cell proliferation at high concentrations (Terao et al. 2001; Horowitz et al. 2007). In our metabolomic analysis, we identified 854 up-regulated and 687 down-regulated differential metabolites in the high *BRCA1* expression cohort. Previous research has highlighted the enrichment of Cysteine and methionine metabolism in oral squamous cell carcinoma and multiple myeloma (Lan et al. 2023; Wang et al. 2021). Protein digestion and absorption have been linked to the progression of osteosarcoma and breast cancer (Shi et al. 2017; Dong et al. 2017). Additionally, D-Amino acid metabolism has been significantly enriched in colorectal cancer (Liu et al. 2022) and carbon metabolism in breast cancer and liver cancer (Cruz-Collazo et al. 2024; Han et al. 2024a, b). Our KEGG enrichment analysis based on metabolomics data indicated that the high-expression cohort of *BRCA1* was notably involved in pathways such as Protein digestion and absorption, Cysteine and methionine metabolism, D-Amino acid metabolism, and Central carbon metabolism in cancer. These findings underscore the connection between disrupted HGSOC vaginal metabolic balance and amino acid metabolic pathways.

Prior studies have indicated the potential of microbiome metabolomics in developing targeted cancer therapies (Xu et al. 2024). Through correlation analysis using a microbial-metabolite interaction network, we found significant negative correlations between *Lactobacillus* and metabolites 5-formamidoimidazole-4-carboxamide ribotide, Cobalt-precorrin-7, INOSINE, and Uridine. Conversely, L-Lysine showed a significant positive correlation with *Lactobacillus*. This suggests that the imbalance in vaginal microbes, particularly the interaction between increased *Lactobacillus* abundance and body metabolite activity, may profoundly affect the host’s regulatory pathway, potentially exacerbating the HGSOC progression. Molecular docking analysis revealed a strong binding potential between 5-formamidoimidazole-4-carboxamide ribotide, negatively correlated with *Lactobacillus* and *METTL1*. This implies that an increase in *Lactobacillus* abundance in high *BRCA1* expression patients may diminish the vaginal content of 5-formamidoimidazole-4-carboxamide ribotide in HGSOC patients, potentially impacting the expression of *METTL1/BRCA1*. These findings offer promising insights for the diagnosis and treatment of HGSOC patients.

To further investigate the function of 5-formamidoimidazole-4-carboxamide ribotide in HGSOC, we conducted in vivo experiments by supplementing the metabolite in nude mouse transplanted tumor models and cancer cells. Our in vitro results demonstrated that supplementation of 5-formamidoimidazole-4-carboxamide ribotide led to significant concentration-dependent inhibition of OVCAR8 cell viability. Moreover, high concentrations of 5-formamidoimidazole-4-carboxamide ribotide promoted apoptosis and decreased the expression of *METTL1*, m7G, and *BRCA1*. Notably, the overexpression of *METTL1* or *BRCA1* reversed the inhibitory effects of 5-formamidoimidazole-4-carboxamide ribotide on OVCAR8 cell proliferation, migration, and invasion. In vivo studies further revealed that 5-formamidoimidazole-4-carboxamide ribotide treatment effectively suppressed tumor growth, with overexpression of *METTL1* or *BRCA1* reversing the inhibitory effects of 5-formamidoimidazole-4-carboxamide ribotide on tumor growth. This study highlights the novel finding that 5-formamidoimidazole-4-carboxamide ribotide suppresses *BRCA1* expression by inhibiting *METTL1*-mediated m7G modification, ultimately leading to the inhibition of HGSOC cell proliferation and tumor growth. Despite these promising results, further research is needed for the clinical translation of 5-formamidoimidazole-4-carboxamide ribotide.

## Conclusions

In summary, our study unveils the association between differences in *BRCA1* expression and changes in vaginal microbiome composition and induced metabolic function in HGSOC patients. In the cohort with high *BRCA1* expression, the abundance of *Lactobacillus* was notably increased, alongside abnormalities in Pantothenate and CoA biosynthesis and amino acid metabolic pathways. We identified 854 up-regulated and 687 down-regulated differential metabolites in the high *BRCA1* expression cohort, suggesting that these dysregulated vaginal microbiota metabolisms may impact HGSOC processes by modulating multiple metabolic pathways. Functionally, we discovered that the metabolite 5-formamidoimidazole-4-carboxamide ribotide inhibits cell proliferation and tumor growth in HGSOC by disrupting *METTL1*-mediated m7G modification of *BRCA1*. This suggests that 5-formamidoimidazole-4-carboxamide ribotide may hold therapeutic potential for the treatment of HGSOC.

## Supplementary Information

Supplementary Material 1: Table S1. Prediction information of m7G modification site of BRCA1 mRNA.

Supplementary Material 2: Figure S1. Full uncropped Blots images in Figures 1-6.

## Data Availability

The data that support the findings of this study are available from the corresponding author upon reasonable request.

## Abbreviations

HGSOC: High-grade serous ovarian carcinoma
OC: Ovarian cancer
PARPi: PolyADP-ribose polymerase inhibitors
*BRCA1*: Breast cancer susceptibility gene 1
*METTL1*: Methyltransferase-like protein 1
m7G: N-7 methylguanosine
RNAseq: RNA sequencing
TCGA: The Cancer Genome Atlas
GTEx: Genotype-Tissue Expression
ASVs: Amplicon Sequence Variants
PCoA: Principal Coordinates Analysis
LDA: Linear Discriminant Analysis
KEGG: Kyoto Encyclopedia of Genes and Genomes
PCA: Principal Component Analysis
PLS-DA: Partial Least Squares Discriminant Analysis
OPLS-DA: Orthogonal Partial Least Squares Discriminant Analysis
CCK-8: Cell Counting Kit-8
RT-qPCR: Real-time quantitative PCR
RIP: RNA Immunoprecipitation
