# Supplementary figures and images for: Microbial metabolite 5-formamidoimidazole-4-carboxamide ribotide targets METTL1 to inhibit m7G modification of BRCA1 mRNA to inhibit high-grade serous ovarian cancer
Source: Mol Med. 2025 Dec 22;32:8. doi: 10.1186/s10020-025-01396-y (PMC12825273; doi:10.1186/s10020-025-01396-y)

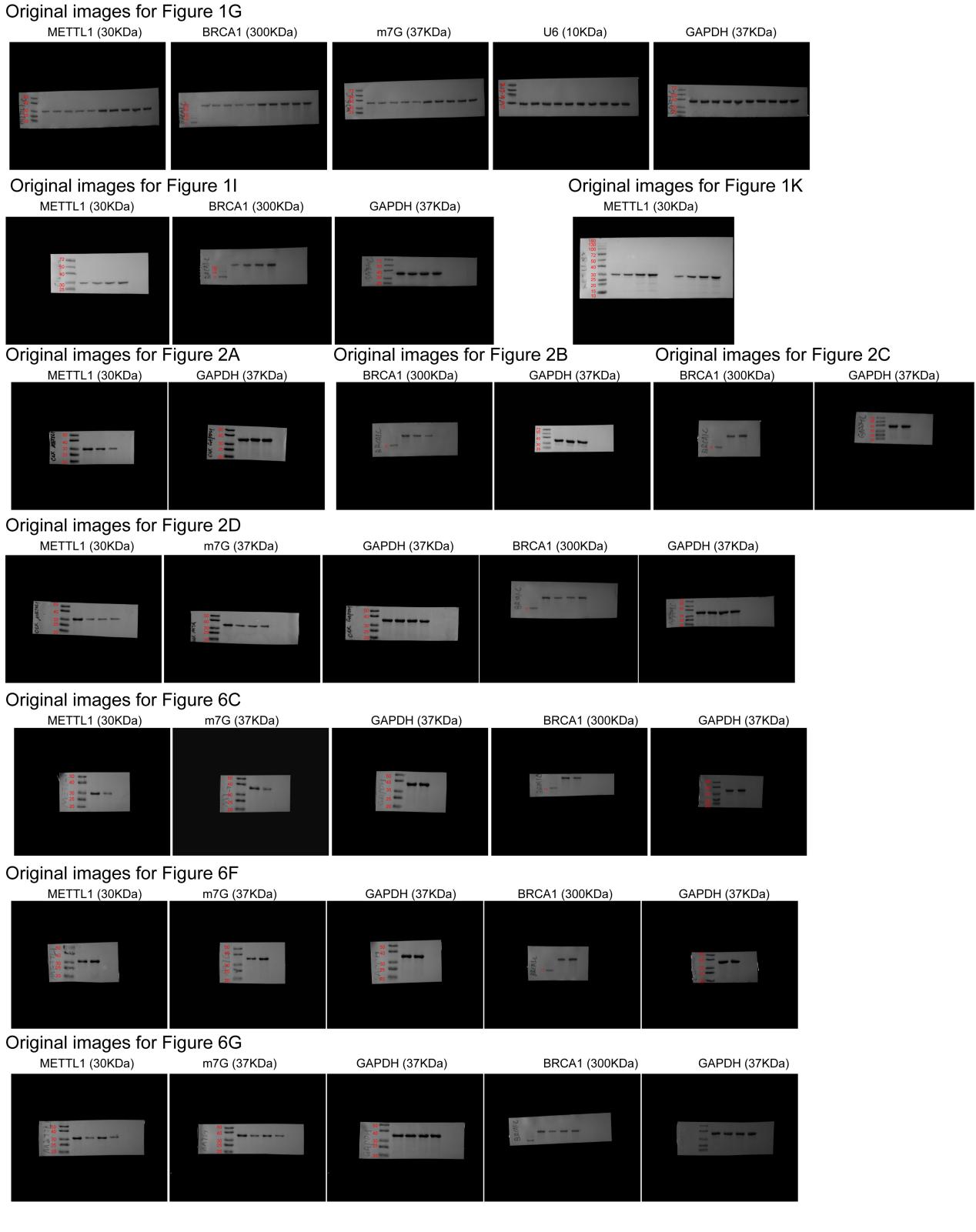


**Figure S1. Full uncropped Blots images in Figures 1-6.**

Supplement: Supplementary file 2 — Supplementary Material 2: Figure S1. Full uncropped Blots images in Figures 1-6. [file 10020_2025_1396_MOESM2_ESM.docx]
